# Cost-Effectiveness of Interventions to Promote Fruit and Vegetable Consumption

**DOI:** 10.1371/journal.pone.0014148

**Published:** 2010-11-30

**Authors:** Linda J. Cobiac, Theo Vos, J. Lennert Veerman

**Affiliations:** School of Population Health, The University of Queensland, Herston, Queensland, Australia; AgroParisTech, France

## Abstract

**Background:**

Fruits and vegetables are an essential part of the human diet, but many people do not consume the recommended serves to prevent cardiovascular disease and cancer. In this research, we evaluate the cost-effectiveness of interventions to promote fruit and vegetable consumption to determine which interventions are good value for money, and by how much current strategies can reduce the population disease burden.

**Methods/Principal Findings:**

In a review of published literature, we identified 23 interventions for promoting fruit and vegetable intake in the healthy adult population that have sufficient evidence for cost-effectiveness analysis. For each intervention, we model the health impacts in disability-adjusted life years (DALYs), the costs of intervention and the potential cost-savings from averting disease treatment, to determine cost-effectiveness of each intervention over the lifetime of the population, from an Australian health sector perspective. Interventions that rely on dietary counselling, telephone contact, worksite promotion or other methods to encourage change in dietary behaviour are not highly effective or cost-effective. Only five out of 23 interventions are less than an A$50,000 per disability-adjusted life year cost-effectiveness threshold, and even the most effective intervention can avert only 5% of the disease burden attributed to insufficient fruit and vegetable intake.

**Conclusions/Significance:**

We recommend more investment in evaluating interventions that address the whole population, such as changing policies influencing price or availability of fruits and vegetables, to see if these approaches can provide more effective and cost-effective incentives for improving fruit and vegetable intake.

## Introduction

Fruits and vegetables are an essential part of the human diet. For adults, a minimum intake of 600 grams per day (approximately equivalent to seven ‘serves’) of fruits and vegetables is estimated to prevent heart disease, stroke and cancer [1], but more than 2.7 million people die each year from inadequate consumption [2]. Reducing this preventable burden of disease by increasing fruit and vegetable intake is one of the key recommendations in the World Health Organisation's Global Strategy on Diet, Physical Activity and Health [3].

In 2004, Pomerleau et al [4] completed a comprehensive review of interventions to promote fruit and vegetable consumption. The review included interventions in supermarkets, worksites, health care settings and the general population as well as interventions targeting specific groups (e.g. low income populations and people with pre-existing disease). They found that the largest increases in fruit and vegetable intake occurred with interventions targeting people with pre-existing disease or disease risk factors (i.e. those at highest risk), but increases of between 0.1 and 1.4 serves of fruits and vegetables per day were observed with interventions targeting the healthy adult population (34 studies) [4]. Interventions using face-to-face counselling were consistently effective, but interventions using less expensive strategies, such as contact via the telephone and computer-tailoring of information, also appeared to be beneficial [4].

To determine which interventions may be good value for money, it is necessary to estimate and compare the ratios of costs to effects for each intervention. Effects can be measured as a change in daily serves or grams of fruits and vegetables, but is more useful to measure intervention effects as a change in disability-adjusted life years (DALYs) or quality-adjusted life years (QALYs), which are derived from an intervention's impact on mortality and morbidity in the population. A cost-effectiveness ratio less than $50,000 (Australia), £30,000 (United Kingdom) or the equivalent of three times the value of per capita gross domestic product (World Health Organisation), per QALY or DALY, is generally considered to be ‘cost-effective’ [5], [6], [7].

To date there have been few economic analyses of interventions to promote fruit and vegetable intake in comparison to the many analyses of interventions targeting other lifestyle risk factors, such as physical activity [8], [9], alcohol consumption [10], [11] and obesity [12], [13]. Of the interventions promoting fruit and vegetable consumption that have been evaluated all have been considered cost-effective (Table 1). However, there are limitations in the evidence of intervention effectiveness underlying these evaluations, with studies lacking a ‘no intervention’ control group for comparison.

**Table 1:** Cost-effectiveness analyses of interventions to promote fruit and vegetable consumption.

Intervention	Cost-effectiveness analysis assumptions	Cost-effectiveness ratio (2003AUD)
‘Fruit n Veg’ community campaign [49] vs. no intervention	Includes intervention costs and disease treatment cost offsets. Intervention effects applied for length of intervention and health effects measured to 15 years. Costs and effects discounted at 3%.	Dominant [50]
‘Fruit n Veg’ community campaign [49] vs. no intervention	Includes intervention costs only. Intervention effects applied for 2 years, with no subsequent relapse and health effects measured to 20 years. Costs and effects discounted at 5%.	$46/QALY(Range: $24/QALY to Dominated) [51]
Behavioural counselling vs. nutritional counselling (low income adults in general practice) [52]	Includes intervention costs only. Intervention effects applied for 1 year, with no subsequent relapse and health effects measured to 20 years. Costs and effects discounted at 5%.	$10,600/QALY(Range: $6,500/QALY to $39,000/QALY) [51]

NB. For comparison, all cost-effectiveness ratios have been adjusted to Australian dollars in the reference year of 2003. An interventions that is ‘Dominant’ leads to more health and less cost, and an intervention that is ‘Dominated’ leads to less health and more costs, than if no intervention to promote fruit and vegetable consumption is in place. QALY – quality adjusted life year.

In this paper, we first update the review of the effectiveness of interventions to promote fruit and vegetable intake, focusing on interventions targeting the healthy adult population (i.e. for primary prevention). We then evaluate the impact of each intervention on population health and calculate cost-effectiveness to determine which (if any) of the interventions would be good value for money for the health sector. We base our analyses of cost-effectiveness in Australia, where 94% of the population eats less than the recommended seven serves of fruits and vegetables each day [14].

## Methods

The cost-effectiveness analysis of interventions to promote fruit and vegetable consumption is one of a series of economic evaluations of interventions to prevent non-communicable disease in the Australian population. To enable future comparison of results, all interventions are being evaluated using the same Assessing Cost-Effectiveness (ACE) method of economic evaluation [15]. This method has been widely used for evaluating interventions and informing policy in an Australian health care context [16], [17], [18].

In this analysis of interventions to promote fruit and vegetable intake, we use the ACE methods to evaluate cost-effectiveness of each program in the Australian population, in comparison to what would occur if no interventions for increasing fruit and vegetable intake were in place. We briefly describe the methods below, but provide further detail of all modelling methods and all input parameter values, sources and assumptions in **Text S1**.

### Interventions

To identify all individual and population-based interventions to increase fruit and vegetable intake in the healthy adult population, we first updated the World Health Organisation review [4] for the period April 2004 to May 2009 using Medline. We excluded studies that (a) were not in English; (b) focused on individuals that were institutionalised or had pre-existing health conditions; (c) were not about fruit and/or vegetables; (d) were not followed up for at least 12 months; (e) evaluated multiple risks without specifying fruit and vegetable intake; (f) did not measure outcomes in grams or serves per day; or (g) did not have a ‘no intervention’ control group for comparison. **Text S2** provides a detailed description of the final 23 interventions included in the cost-effectiveness analysis.

For cost-effectiveness analysis, we first identify the key components of each intervention and characteristics of the target group (e.g. age, sex). We then quantify the type and number of resources used in recruitment of intervention participants, intervention delivery and participation; and combined this information with estimates of resource costs in Australia, to determine a cost per participant for each intervention if delivered to the relevant target group in the Australian population (**Text S3**). All intervention effects on consumption of fruits and vegetables are translated into serves per day of fruits and vegetables, combined, assuming one serve is equivalent to 80 g where definition is not provided in the study.

### Health outcomes

Analysis of National Health Survey data shows that the current consumption of fruits and vegetables in Australia is positively skewed [19]. As has been found with data from the United States [1] and the Netherlands [20], we found that the Australian fruit and vegetable consumption data is best described by a Weibull distribution. We assume that each intervention changes the mean value of the Weibull distribution, and that there is no excess risk of disease for adults consuming at least 600 mg/day [1] of fruits and vegetables.

We combine the Weibull distributions of fruit and vegetable consumption, before and after the intervention, with known risks of disease related to fruit and vegetable exposure (Table 2) to determine the population impact fraction [21] of each intervention on the incidence of ischaemic heart disease and stroke, and colon, lung, stomach and oesophageal cancers. We also evaluate the maximum health gain that could be achieved if all adults consumed at least 600 mg/day of fruits and vegetables.

**Table 2:** Unit change in relative risk with an 80 gram per day increase in consumption of fruits and vegetables (approximately equivalent to one serve) [1].

	15–69 years	70–79 years	80+ years
Ischaemic heart disease	0.9 (0.82–0.99)	0.93 (0.85–1.01)	0.95 (0.87–1.03)
Ischaemic stroke	0.94 (0.89–0.99)	0.95 (0.91–1)	0.97 (0.92–1.02)
Lung cancer	0.96 (0.93–0.99)	0.97 (0.91–1.02)	0.98 (0.92–1.03)
Stomach cancer	0.94 (0.86–1.03)	0.95 (0.87–1.04)	0.97 (0.89–1.06)
Oesophageal cancer	0.94 (0.88–1.01)	0.95 (0.89–1.02)	0.97 (0.91–1.04)
Colon cancer	0.99 (0.97–1.02)	0.99 (0.97–1.02)	1 (0.97–1.02)

NB. Values are mean and 95% confidence interval. We assume no excess risk of disease for adults consuming at least 600 mg/day [1] of fruits and vegetables.

Change in prevalence and mortality of each disease and change in total years of life lived are derived for every five-year age group cohort in the adult population (age 18 years and older) using proportional multi-state lifetable analysis [22]. The years of life lived are then adjusted for time spent in ill health (‘disability’) to determine disability-adjusted life years (DALYs) averted.

All disease incidence and case fatality rates and the prevalence of years lived with disability in Australia are obtained from the Australian Burden of Disease and Injury study sources [23], using the DISMOD II analysis tool [24] to derive rates not explicitly reported. Trends in disease incidence and case fatality were applied for the first 20 years, based on Australian projections [25], with rates assumed to remain constant thereafter.

### Costs

We calculate the total cost of intervention to Government and patients, including patient time and travel costs, from the intervention cost per participant and total number of participants recruited from the Australian population. Australian rates of recruitment are assumed to be similar to those observed in the interventions studies. We exclude one-off intervention start-up costs (e.g. for research and development of intervention materials) so that cost-effectiveness of interventions are compared as if running in ‘steady-state’.

We also calculate health care costs that could be averted with intervention, through not having to treat as many cases of the diseases related to fruit and vegetable exposure. Cost per prevalent case of ischaemic heart disease and stroke, and cost per incident case of colon, lung, stomach and oesophageal cancers, are derived from Australia's Disease Costs and Impacts Study data [26]. All costs are converted to Australian dollars in the reference year of 2003 using the relevant Australian Consumer Price Index [27] or Health Price Index [28].

### Cost-effectiveness modelling

We model each intervention as a one-off event in the Australian population in the baseline year of 2003. We assume that effects on consumption are sustained for the duration of the intervention (between one and five years), but there is only weak evidence around the long-term sustainability of lifestyle behavioural changes in the population thereafter. In two of the fruit and vegetable intervention trials with follow-up of participants at one year and again at two years, the mean intervention effect on fruit and vegetable intake measured at the end of the intervention (i.e. at one year) had decreased by 62% [29] and 50% [30] by the end of the subsequent year. In the cost-effectiveness analysis, we assume that effects on consumption decrease by 50% per year after the end of the intervention, but evaluate sensitivity of the results to variations in the rate of decay between 0% and 100%. We also evaluate sensitivity of the results to removing the future trends in disease incidence and case fatality, and to a halving or doubling of the 80 g estimate of serving size for the intervention trials.

To determine the cost-effectiveness of each intervention, we measure all costs and effects over the lifetime of the population, discounting future costs and DALYs at a rate of 3% [31], to calculate a cost-effectiveness ratio in Australian dollars per DALY averted. We use Monte Carlo analysis (@Risk; Palisade, Version 4.5) to derive 95% uncertainty intervals for all outcome measures and to determine probabilities of intervention cost-effectiveness against a cost-effectiveness threshold of A$50,000 per DALY [6], [8], [32].

## Results

Twenty-three interventions for promoting fruit and vegetable intake meet the evidence criteria for cost-effectiveness analysis: eight interventions in the general population, one supermarket intervention, seven worksite interventions, three interventions in health care settings and four interventions targeting low-income populations. Most rely on multiple strategies to promote fruit and vegetable intake, such as counselling, mail-out of information, hand-out of promotional material, financial incentives and special promotional events (Table 3). The availability of evidence for evaluation of intervention effects is limited and of variable strength, with studies ranging from properly designed randomised controlled trial (e.g. dietary counselling trial [33]) to before-after survey in intervention and comparison communities (e.g. community intervention program [34]).

**Table 3:** Summary of key intervention components, cost per participant, the number of participants recruited (if rolled out in Australia), the net intervention effect on fruit and vegetable consumption and strength of evidence underlying the measure of effect.

	Key intervention components	Cost per participant	Number of participants	Net effect (serves/day)*	Strength of evidence**
**General population**					
Marcus 1998 [37]	Telephone counselling; information mail-out	$42	515	0.39 (0.15)	Limited (III-1)
Radakovich 2006 [53]	Individual dietary counselling	$1,280	23,958	6.2 (0.53)	Limited (III-1)
Howard 2006 [33]	Individual and group dietary counselling	$1,519	17,086	1.2 (0.02)	Limited (II)
Heimendinger 2005a [35]	Information mail-out (tailored)	$8	515	0.17 (0.14)	Limited (III-1)
Heimendinger 2005b [35]	Information mail-out (multiple tailored)	$10	515	0.38 (0.14)	Limited (III-1)
Heimendinger 2005c [35]	Information mail-out (multiple re-tailored)	$10	515	0.45 (0.14)	Limited (III-1)
Greene 2008 [29]	Phone counselling; information mail-out	$756	448,208	0.65 (0.24)	Limited (III-1)
Ashfield-Watt 2007 [34]	Community-based events, sponsorship, promotion	$3	15,083,863	0.30 (0.16)	Limited (III-3)
**Supermarket**					
Kristal 1997 [38]	Supermarket displays, flyers, discount coupons	$45	3,340,087	0.030 (0.19)	Limited (III-3)
**Worksite**					
Tilley 1999 [30]	Information seminars; promotional materials	$122	538,898	0.20 (0.06)	Limited (III-1)
Hebert, 1993 [39]	Information seminars; promotional materials; cafeteria changes	$2,700	538,898	0.29 (0.12)	Limited (III-2)
Sorensen 1996 [54]	Information seminars; promotional materials; cafeteria changes	$240	538,898	0.18 (0.05)	Limited (III-3)
Emmons 1999 [55]	Information seminars; promotional materials; cafeteria changes	$303	538,898	0.40 (0.08)	Limited (III-3)
Sorensen 1998 [40]	Information seminars; promotional materials; cafeteria changes	$240	538,898	0.13 (0.06)	Limited (III-3)
Beresford 2001 [56]	Information seminars; promotional materials; cafeteria changes	$192	538,898	0.29 (0.15)	Limited (III-1)
Engbers 2006 [36]	Promotional materials; cafeteria changes	$110	538,898	2.5 (5.9)	Limited (III-2)
**Health care setting**					
Kristal 2000 [57]	Telephone counselling, information mail-out	$202	71,207	0.33 (0.10)	Limited (III-1)
Stevens 2003 [58]	Dietary counselling, telephone follow-up	$297	11,443	1.1 (0.16)	Limited (III-1)
Sacerdote 2006 [59]	Dietary counselling, information mail-out	$94	534,598	0.19 (0.24)	Limited (II)
**Low income**					
Nitzke 2007 [42]	Telephone counselling; information mail-out	$333	6,412	0.38 (0.25)	Limited (III-1)
Herman 2008a [60]	Farmers' market vouchers	$357	24,792	2.6 (0.85)	Limited (III-2)
Herman 2008b [60]	Supermarket vouchers	$357	24,792	1.1 (0.89)	Limited (III-2)
Havas 2003 [41]	Peer counselling; telephone counselling; promotional materials	$1,600	93,724	0.42 (0.19)	Limited (III-1)

*Mean and standard error.

**Strength of evidence for cost-effectiveness analysis classified as ‘Sufficient’, ‘Limited’ or ‘Inconclusive’, based primarily on NHMRC [61] levels of evidence (in brackets). Full details of classification system are provided in **Text S4**.

Overall, there are 110,000 DALYs (95% uncertainty interval: 59,000 to 150,000) attributable to insufficient consumption of fruits and vegetables. The community intervention program [34] is the most effective intervention, but has the potential to avert only 4.9% of the total burden (Table 4). The majority of interventions (21 out of the 23) could avert less than 1% of the total health burden.

**Table 4:** Health gain, costs and cost-effectiveness of the interventions to promote fruit and vegetable consumption.

	Mean DALYs averted	Proportion of total DALYs	Mean intervention cost ($million)	Mean disease cost offset ($million)	Median CER ($/DALY)	Probability (<$50/000/DALY)
**General population**						
Marcus 1998	0.23 (0.06 to 0.46)	<0.01%	$0.02 ($0.02 to $0.03)	$0.002 ($0.005 to $0.001)	$84,000	12%
Radakovich 2006	33 (0.34 to 140)	0.03%	$32 ($0.41 to $130)	$0.32 ($1.4 to $0.00)	$950,000	0%
Howard 2006	85 (6.3 to 280)	0.08%	$25 ($2.0 to $84)	$0.76 ($2.7 to $0.06)	$280,000	0%
Heimendinger 2005a	0.10 (−0.07 to 0.29)	<0.01%	$0.004 ($0.003 to $0.005)	$0.001 ($0.003 to $0.001)	$27,000	68%
Heimendinger 2005b	0.22 (0.05 to 0.45)	<0.01%	$0.005 ($0.004 to $0.007)	$0.002 ($0.005 to $0.001)	$12,000	95%
Heimendinger 2005c	0.27 (0.09 to 0.50)	<0.01%	$0.005 ($0.004 to $0.007)	$0.003 ($0.006 to $0.001)	$8,600	98%
Greene 2008	760 (180 to 1,500)	0.72%	$340 ($150 to $620)	$8.4 ($16 to $2.0)	$420,000	0%
Ashfield-Watt 2007	5,200 (−430 to 11,000)	4.9%	$47 ($29 to $78)	$54 ($130 to $4.2)	Dominant	94%
**Supermarket**						
Kristal 1997	100 (−1,400 to 1,600)	0.10%	$150 ($120 to $180)	$1.0 ($16 to $14)	$2,500,000	0.2%
**Worksite**						
Tilley 1999	100 (16 to 310)	0.09%	$66 ($17 to $150)	$0.98 ($2.9 to $0.15)	$670,000	0%
Hebert, 1993	230 (19 to 730)	0.21%	$1,500 ($690 to $2,700)	$2.2 ($7.3 to $0.16)	$7,400,000	0%
Sorensen 1996	180 (30 to 500)	0.17%	$130 ($77 to $190)	$1.7 ($4.8 to $0.28)	$790,000	0%
Emmons 1999	540 (96 to 1,500)	0.50%	$160 ($100 to $240)	$5.1 ($14 to $0.85)	$320,000	0%
Sorensen 1998	130 (9.1 to 430)	0.12%	$130 ($77 to $190)	$1.2 ($4.0 to $0.09)	$1,200,000	0%
Beresford 2001	280 (−24 to 970)	0.26%	$100 ($61 to $170)	$2.7 ($9.2 to $0.24)	$430,000	0%
Engbers 2006	1,200 (−5,300 to 9,600)	1.2%	$60 ($21 to $130)	$12 ($92 to $54)	$47,000	50%
**Health care setting**						
Kristal 2000	21 (1.3 to 75)	0.02%	$14 ($8.4 to $28)	$0.21 ($0.75 to $0.01)	$900,000	0%
Stevens 2003	17 (1.3 to 59)	0.02%	$3.4 ($0.25 to $11)	$0.16 ($0.56 to $0.01)	$180,000	0%
Sacerdote 2006	96 (−120 to 370)	0.09%	$50 ($26 to $80)	$0.93 ($3.7 to $1.2)	$530,000	0%
**Low income**						
Nitzke 2007	0.20 (−0.04 to 1.1)	<0.01%	$2.2 ($0.02 to $10)	$0.002 ($0.009 to $0.000)	$10,000,000	0%
Herman 2008a	32 (9.6 to 61)	0.03%	$8.8 ($7.2 to $11)	$0.32 ($0.63 to $0.08)	$270,000	0%
Herman 2008b	13 (−8.0 to 39)	0.01%	$8.8 ($7.2 to $11)	$0.14 ($0.40 to $0.08)	$660,000	0%
Havas 2003	35 (2.1 to 79)	0.03%	$150 ($120 to $190)	$0.34 ($0.79 to $0.02)	$4,400,000	0%

NB. All values are rounded to two significant figures. Costs are in Australian dollars referenced to the year 2003. The 95% uncertainty interval is presented for all disability-adjusted life years (DALYs) and costs. Where the cost-effectiveness ratio (CER) is ‘Dominant’, the intervention leads to more health and less cost than if no fruit and vegetable intervention is in place. Results are discounted at 3% (undiscounted values are presented in Text S5).

Five out of the 23 interventions have a median cost-effectiveness ratio under a $50,000 per DALY threshold. The community intervention program [34] is most cost-effective, with potential to increase population health at a cost-saving to the health sector. The three lowest cost interventions [35] are also cost-effective. These interventions are based on mail-out of tailored information to intervention participants, but while this approach keeps costs low, the potential population health benefit from any of these interventions is very small (less than one DALY) because participants are recruited only from callers to a Cancer information service and the proportion of callers who are eligible (e.g. not receiving or awaiting cancer treatment) and willing to participate in the trial was small (11%). The worksite health promotion intervention from the Netherlands [36] also has a median cost-effectiveness ratio under $50,000 per DALY, but there is substantial uncertainty around intervention effectiveness, leading to only a 50% probability that this intervention will be cost-effective if rolled out in Australia.

Intervention cost-effectiveness ratios are slightly more favourable when not discounted, due to greater intervention health gains and cost offsets (Text S5). However, only one additional intervention, a low cost telephone counselling intervention [37], falls under the cost-effectiveness threshold. If we remove the predicted trends in disease incidence and case fatality, intervention cost-effectiveness ratios become slightly more favourable, but the difference is small and the overall result (five out of 23 interventions cost-effective) remains the same. Cost-effectiveness ratios become less favourable with decreases in serving size and more favourable with increases in serving size, but even with a 100% increase in serving size, only three additional interventions are considered cost-effective against the $50,000 per DALY threshold.

The results are somewhat more sensitive to how quickly we assume the intervention effects on fruit and vegetable consumption diminish over time (Table 5). Six of the interventions, an individual dietary counselling intervention [33], the supermarket intervention [38], two worksite intervention programs [39], [40] and two counselling interventions for low income populations [41], [42] are not cost-effective under any decay scenario. Overall, under the worst-case scenario (no health benefits sustained beyond the end of intervention), none of the 23 interventions are cost-effective. Under the best-case scenario (health benefits sustained for life after one-off intervention) 17 interventions are cost-effective, although this scenario seems very unlikely.

**Table 5:** Sensitivity of intervention cost-effectiveness to rates of decay in intervention effectiveness between 0% (life-long health benefits from one-off intervention) and 100% (no health benefits beyond the end of intervention).

	Median cost-effectiveness ratio (A$/DALY)				
	0% decay	25% decay	50% decay*	75% decay	100% decay
**General population**					
Marcus 1998 [37]	Dominant	$24,000	$84,000	$260,000	Dominated
Radakovich 2006 [53]	$5,100	$290,000	$950,000	$2,900,000	Dominated
Howard 2006 [33]	$71,000	$210,000	$280,000	$330,000	$350,000
Heimendinger 2005a [35]	Dominant	$3,800	$27,000	$99,000	Dominated
Heimendinger 2005b [35]	Dominant	Dominant	$12,000	$55,000	Dominated
Heimendinger 2005c [35]	Dominant	Dominant	$8,600	$43,000	Dominated
Greene 2008 [29]	$33,000	$160,000	$420,000	$1,200,000	Dominated
Ashfield-Watt 2007 [34]	Dominant	Dominant	Dominant	$14,000	Dominated
**Supermarket**					
Kristal 1997 [38]	$95,000	$890,000	$2,500,000	$7,100,000	Dominated
**Worksite**					
Tilley 1999 [30]	$11,000	$220,000	$670,000	$2,000,000	Dominated
Hebert, 1993 [39]	$340,000	$3,500,000	$7,400,000	$10,000,000	$11,000,000
Sorensen 1996 [54]	$40,000	$400,000	$790,000	$1,200,000	$1,600,000
Emmons 1999 [55]	$17,000	$180,000	$320,000	$400,000	$430,000
Sorensen 1998 [40]	$67,000	$630,000	$1,200,000	$1,900,000	$2,500,000
Beresford 2001 [56]	$17,000	$210,000	$430,000	$650,000	$870,000
Engbers 2006 [36]	Dominant	$10,000	$47,000	$150,000	Dominated
**Health care setting**					
Kristal 2000 [57]	$18,000	$300,000	$900,000	$2,700,000	Dominated
Stevens 2003 [58]	Dominant	$58,000	$180,000	$570,000	Dominated
Sacerdote 2006 [59]	$6,000	$170,000	$530,000	$1,600,000	Dominated
**Low income**					
Nitzke 2007 [42]	$57,000	$3,700,000	$10,000,000	$32,000,000	Dominated
Herman 2008a [60]	$1,000	$91,000	$270,000	$810,000	Dominated
Herman 2008b [60]	$15,000	$230,000	$660,000	$1,900,000	Dominated
Havas 2003 [41]	$290,000	$2,300,000	$4,400,000	$6,400,000	$7,700,000
Number of interventions:					
Cost-effective**	17	6	5	2	0
Cost-saving***	7	3	1	0	0

*Base-case assumption.

**Cost-effectiveness ratio less than A$50,000/DALY.

***Dominant cost-effectiveness ratio.

## Discussion

Overall, the interventions evaluated for promoting fruit and vegetable consumption have low potential for improving population health, have relatively high costs and are mostly not cost-effective strategies for health sector investment. Out of 23 interventions, only five are cost-effective, and even the most effective intervention could avert only 5% of the disease burden attributable to insufficient intake of fruits and vegetables.

Previous evaluations (Table 1) have identified two interventions that are cost-effective for promoting fruit and vegetable intake. However, the results are not directly comparable to our cost-effectiveness ratios due to differences in analysis techniques and assumptions (e.g. discount rates). The previous analyses were based on intervention evaluation studies that did not include measurement in a comparison region that was not exposed to intervention and were, therefore, ruled out of our analyses. In taking a systematic approach to evaluating all fruit and vegetable interventions that meet stronger evidence inclusion criteria, we have shown that while a few interventions may be cost-effective, the vast majority of current intervention approaches are not a good investment for the health sector. Had there been more homogeneity in the delivery components and target groups for the interventions, so that we could take the more usual cost-effectiveness approach of pooling interventions of the same type and evaluating cost-effectiveness from the pooled intervention effect and modal intervention cost, none of the interventions for promoting fruits and vegetables would be cost-effective.

Other interventions that we did not evaluate due to insufficient evidence, such as changes to policies that influence availability of or access to affordable fruits and vegetables or lead to benefits indirectly (e.g. through taxing unhealthy foods), may be more cost-effective in promoting fruit and vegetable intake. Interventions that can shift the distribution of a risk factor in the whole population, as Geoffrey Rose [43] advocated, are generally more effective for improving population health than interventions targeting high-risk individuals. In a country like Australia, where 94% of the population are at increased risk of disease due to insufficient fruit and vegetable intake [14], interventions that are aimed at the whole population are a logical strategy for reducing disease burden.

The community intervention program [34] was the only intervention program targeting a whole community or population that met our evidence criteria for cost-effectiveness analysis. This program, which was developed under the ‘five a day’ initiative in the United Kingdom (UK), is run by a board of local community representatives, who aim to engage local community groups and retailers in delivering intervention strategies suitable to the local area (e.g. community gardens, school promotion activities, sponsorship of sporting teams, home grocery delivery, etc.). In our analysis, this intervention was found to be the most effective and cost-effective approach to promoting fruit and vegetable intake. However, the evidence underlying the measure of intervention effect was relatively weak making it difficult to rule out bias and/or confounding of intervention effects. In the UK evaluation, the intervention appeared to arrest a decline in fruit and vegetable intake observed in the non-exposed population, but differences between the exposed and non-exposed populations (e.g. smoking) somewhat biased the measure of effect on fruit and vegetable intake [34].

### Limitations to modelling

Lack of data on the sustainability (or maintenance) of behavioural changes is the key unknown in modelling cost-effectiveness of preventive interventions. Our assumption of an exponential decay in effect at the rate of 50% per year is comparable to the pattern of weight regain derived from meta-regression of diet and exercise trials for weight loss [44]. It also matches the amount of first year decay observed in one of the fruit and vegetable worksite intervention trials [30], but is slightly more optimistic that the amount of first year decay observed in one of the phone counselling trials [29]. However, different intervention components (e.g. phone counselling versus face-to-face counselling) may also lead to differential rates of decay between interventions. Overall, a more rapid loss of behaviour change in the population would reduce the preventive effects on disease morbidity and mortality over the lifetime, and lead to fewer cost-effective interventions, and *vice versa*. Only with more long-term follow-up in intervention studies (e.g. beyond 12 months) will it be possible to assess the accuracy of our current estimates on the maintenance of behaviour changes following intervention.

We also did not explicitly model the health consequences of any change in fat intake associated with the intervention effects on fruit and vegetable consumption. The relationship between micro-nutrient food components (e.g. beta-carotene), macro-nutrient components (e.g. fat), food groups (e.g. fruits and vegetables) and disease outcomes is complex and still quite poorly understood. While fat intake has been measured in some intervention trials, it is in all cases a measure of total fat (or percentage of energy intake from total fat) and not broken down into what we now believe to be the potentially harmful fats (e.g. saturated fats and trans fats) and potentially beneficial fats (e.g. monounsaturated and especially polyunsaturated fats). In this paper we chose to focus on the broader food group of fruits and vegetables, for which we have the most concrete evidence, and not attempt to explicitly model the different micro- and macro-nutrient pathways to disease outcomes, but it is possible that we underestimate the health impacts of the interventions.

Prevention of disease inevitably leads to added years of life, mostly at older age when morbidity is higher [45]. Incorporating the costs of health care for diseases other than those primarily modelled will alter cost-effectiveness ratios and can influence intervention rankings, particularly where interventions target different age groups [46]. We do not include these costs in our baseline analyses of cost-effectiveness to ensure results for the fruit and vegetable interventions are comparable to other interventions evaluated for the Australian health care system using the ACE method of economic evaluation [15]. When we add the health care costs of unrelated disease in added years of life into our evaluation of the fruit and vegetable interventions (results not shown), we get even less favourable cost-effectiveness ratios. Overall, one less intervention (the worksite intervention of Engbers 2006 [36]) is cost-effective against the $50,000/DALY threshold.

It would be possible to simulate an intervention that is repeated at regular intervals. Given the lack of evidence around behaviour changes with repeated interventions, however, we would need to make a number of additional assumptions. For example, we would need to consider how often the intervention program should be repeated (e.g. 1 year, 2 years, 5 years?), and we would need to estimate how well the effectiveness of the program is sustained when received for the second, third fourth, etc. time. These additional analyses would also need to be subjected to sensitivity analyses. Overall, we do not think this would provide more realistic outcomes about the cost-effectiveness of the fruit and vegetable interventions or more clarity about how sensitive they are to the assumptions that are made.

### Policy implications

While cost-effectiveness has been evaluated in the context of the Australian health care system, using Australian rates of disease, costs of intervention resources and costs of disease treatment, it is likely that the results would be broadly applicable to other countries with a similar epidemiological profile. North American and Western European countries have a similar proportion of disease burden attributable to insufficient fruits and vegetables [2].

Given the high population prevalence of insufficient fruit and vegetable intake in Western countries, further research is needed to identify more effective and cost-effective approaches to promoting fruit and vegetable intake. Population-targeted approaches may have potential to achieve larger reductions in the disease burden attributable to insufficient fruit and vegetable intake, but it is imperative that these programs receive sufficient funding to properly monitor and evaluate outcomes.

Compared to interventions targeting individual behaviour, interventions exposing whole communities or populations to incentives to change are less amenable to evaluation in the traditional controlled trial [47]. The weaker study designs associated with population-targeted interventions increase the risk of being unable to reproduce health outcomes when implementing interventions on a broader scale. Health care decision-makers must therefore weigh up the uncertainty in outcomes against the potential size of return in population health improvements (or reductions in health expenditure). Although decision-makers in health care are known to be highly risk averse [48], in the case of these preventive types of interventions to promote fruits and vegetables, the probability of serious unintended outcomes (e.g. loss of life) is very low.

## Supporting Information

Text S1 Modelling methods and input data.
(0.40 MB DOC)

Text S2 Intervention descriptions.
(0.14 MB DOC)

Text S3 Intervention resource cost components.
(0.14 MB DOC)

Text S4 Evidence classification table.
(0.02 MB DOC)

Text S5 Table of undiscounted results.
(0.08 MB DOC)
