# Supplementary material for: Cost-Effectiveness of Interventions to Promote Fruit and Vegetable Consumption
Source: PLoS One. 2010 Nov 30;5(11):e14148. doi: 10.1371/journal.pone.0014148 (PMC2994753; doi:10.1371/journal.pone.0014148)
Supplement: Text S1 — Modelling methods and input data. (0.40 MB DOC) [file pone.0014148.s001.doc]

**Cost-effectiveness modelling methods**

A model was developed in Excel to evaluate the cost-effectiveness of interventions to promote fruit and vegetable consumption. The model evaluates intervention cost-effectiveness over the lifetime of the Australian population in a baseline year of 2003, using information on intervention costs and effects from the fruit and vegetable intervention literature and Australian data on disease epidemiology and costs of disease treatment. Uncertainty analysis is carried out using the add-in tool @Risk (Palisade, Version 4.5).

**Life table analysis**

To determine the health benefits of an intervention, we first calculate the difference in years of life lived between an Australian population that continues to consume fruits and vegetables at current rates and an identical population that receives the intervention. Each population is divided into five-year age group cohorts (from age 15-19 to age 95+), and simulated in a proportional multi-state lifetable [1] (Figure 1) until everyone has either died or reached 100 years of age.

The years of life lived by each cohort are adjusted at each age for time spent in poor health (‘disability’) due to disease or injury, using disability estimates from the Australian Burden of Disease study [2,3]. For modelled disease, the disability adjustment is calculated as the number of prevalent years lived with disability (pYLD) per prevalent case of disease. The age- and sex-specific probability of health loss due to disability from all other causes (i.e. those not specifically modelled) is calculated as the number of pYLD per capita for these conditions. The final difference in disability-adjusted life years (DALYs) is the measure of health gain attributed to an intervention.

**Figure 1:** Schematic of a proportional multi-state lifetable, showing the interaction between disease parameters and lifetable parameters, where x is age, i is incidence, p is prevalence, m is mortality, w is disability-adjustment, q is probability of dying, l is number of survivors, L is life years, Lw is disability-adjusted life years and HALE is health-adjusted life expectancy, and where ‘-‘ denotes a parameter that specifically excludes modelled diseases, and ‘+’ denotes a parameter for all diseases (i.e. including modelled diseases).

**Disease models**

Diets low in fruits and vegetables have been linked to ischaemic heart disease and stroke, and colon, lung, stomach and oesophageal cancers [4]. Each of these diseases is modelled explicitly using a set of differential equations that describe the transition of people between four states (healthy, diseased, dead from the disease, and dead from all other causes), with transition of people between the four states based on rates of mortality, incidence, case fatality and remission (Figure 2) [5].

Epidemiological data inputs to the disease models are derived from the Australian Burden of Disease study [2,3], with the aid of the epidemiological analysis tool DISMOD II ([www.epigear.com](../www.epigear.com)) to derive data not explicitly reported (e.g. case fatality and prevalence from incidence and mortality rates). Future changes in disease incidence and case fatality are based on twenty-year predictions in the Australian Burden of Disease study, with rates assumed to remain constant thereafter.

Healthy

Diseased

Dead

(disease)

Dead

(other)

Mortality

(other)

Remission

Incidence

Case

fatality

Mortality (other)

Figure 2: Each disease in the physical activity analysis is modelled by a conceptual model with four states (healthy, diseased, dead from the disease, and dead from all other causes) and transition hazards between states of incidence, remission, case fatality and mortality from all other causes (after Barendregt et al. [4])

**Intervention effects**

The health benefits of interventions to promote fruit and vegetable consumption are modelled via a reduction in incidence of each modelled disease (Equation 1).

$\text{I}' = \text{I} \times\left(1 - \text{PIF}\right)$ (1)

where:

$\text{I}$ is the incidence of the disease in the population (by age and sex);

$\text{I}'$ is the new incidence of the disease after an intervention is implemented; and

$\text{PIF}$ is the potential impact fraction for the intervention.

Each PIF [6] is derived from three key parameters: the current distribution of fruit and vegetable consumption in the Australian population, the new distribution of fruit and vegetable consumption in the Australian population *after* an intervention is implemented, and the relative risk of disease (Equation 2).

(2)

where:

RR(*x*) is the distribution function for the relative risk of ischaemic heart disease, ischaemic stroke, colon cancer, lung cancer, stomach cancer or oesophageal cancer (Table 1);

p(*x*) is the current prevalence distribution of fruit and vegetable consumption;

p′(*x*) is the prevalence distribution of fruit and vegetable consumption after an intervention is implemented; and

*a* and *b* are the lower and upper bounds of the integration (we assume *a*= 0 g/day and *b* = 2,000 g/day)*.*

Table 1: Unit change in relative risk with an 80 gram per day increase in consumption of fruits and vegetables (approximately equivalent to one serve) [4].

|  | **15-69 years** | **70-79 years** | **80+ years** |
| --- | --- | --- | --- |
| Ischaemic heart disease | 0.9 (0.82 – 0.99) | 0.93 (0.85 – 1.01) | 0.95 (0.87 – 1.03) |
| Ischaemic stroke | 0.94 (0.89 – 0.99) | 0.95 (0.91 – 1) | 0.97 (0.92 – 1.02) |
| Lung cancer | 0.96 (0.93 – 0.99) | 0.97 (0.91 – 1.02) | 0.98 (0.92 – 1.03) |
| Stomach cancer | 0.94 (0.86 – 1.03) | 0.95 (0.87 – 1.04) | 0.97 (0.89 – 1.06) |
| Oesophageal cancer | 0.94 (0.88 – 1.01) | 0.95 (0.89 – 1.02) | 0.97 (0.91 – 1.04) |
| Colon cancer | 0.99 (0.97 – 1.02) | 0.99 (0.97 – 1.02) | 1 (0.97 – 1.02) |
| NB. Values are mean and 95% confidence interval. We assume no excess risk of disease for adults consuming at least 600 mg/day [4] of fruits and vegetables. | | | |

Analysis of National Health Survey [7] data shows that the current consumption of fruits and vegetables in Australia is positively skewed. As has been found with data from the United States [4] and the Netherlands [8], we found that the Australian fruit and vegetable consumption data is best described by a Weibull distribution. This is illustrated for 45 to 49 year olds in Figure 1. Each Weibull distribution is defined by a shape (α) and scale (β) parameter (Table 2). The mean (Equation 3) and standard deviation (Equation 4) can be derived from the Weibull parameters and the gamma function (Equation 5). We assume that each intervention increases (or decreases) the mean value of the Weibull distribution.

$\mu= \frac{\beta}{\alpha} \Gamma\left(\frac{1}{\alpha}\right)$ (3)

$\sigma= \beta\left[\sqrt{\Gamma\left(\frac{1}{2 \alpha}\right)} - \Gamma\left(\frac{1}{\alpha}\right)\right]$ (4)

$\Gamma\left(x\right) = \int_{0}^{\infty} e^{-t} t^{x - 1} dt$ (5)

Figure 1: Weibull distributions fitted to National Health Survey 2001 measures of fruit and vegetable consumption, illustrated for 45-49 year old men and women.

Table 2: Weibull distribution parameters (α = shape and β = scale) by age and sex.

| **Age (years)** | **Men** | | **Women** | |
| --- | --- | --- | --- | --- |
|  | **α** | **β** | **α** | **β** |
| 15-19 | 1.8 | 334 | 2.2 | 365 |
| 20-24 | 1.8 | 299 | 1.9 | 346 |
| 25-29 | 1.9 | 300 | 2.1 | 361 |
| 30-34 | 1.9 | 330 | 2.2 | 384 |
| 35-39 | 2.0 | 336 | 2.3 | 384 |
| 40-44 | 1.9 | 349 | 2.4 | 407 |
| 45-49* | 1.9 | 352 | 2.3 | 407 |
| 50-54 | 2.0 | 378 | 2.5 | 446 |
| 55-59 | 2.2 | 395 | 2.7 | 456 |
| 60-64 | 2.3 | 421 | 2.6 | 470 |
| 65-69 | 2.4 | 431 | 2.7 | 452 |
| 70-74 | 2.7 | 430 | 3.1 | 458 |
| 75-79 | 2.6 | 432 | 3.0 | 450 |
| 80-84 | 2.7 | 426 | 2.9 | 435 |
| 85+ | 2.8 | 416 | 2.8 | 411 |
| * Illustrated in Figure 1. | | | | |

**Disease costs**

Cost offsets, due to reduced rates of diseases related to fruit and vegetable consumption, are evaluated using disease treatment costs from the Australian Institute of Health and Welfare Disease Costs and Impacts Study 2001 (Table 3). The average costs per prevalent case of ischaemic heart disease and stroke and per incident case of cancer are derived from the Australian prevalence of disease in 2001 [3], adjusted to the year 2003 using the Australian Health Price Index [9].

Table 3: Cost per prevalent case of ischaemic heart disease and stroke and per incident case of colon, lung, stomach or oesophageal cancer.

| **Age (years)** | **Ischaemic heart disease*** | **Ischaemic stroke*** | **Colon cancer**** | **Lung cancer**** | **Stomach cancer**** | **Oesophageal cancer**** |
| --- | --- | --- | --- | --- | --- | --- |
| Men |  |  |  |  |  |  |
| <55 | $2,962 | $2,228 | $17,490 | $17,181 | $22,268 | $28,939 |
| 55–64 | $1,988 | $4,942 | $17,657 | $14,765 | $20,992 | $29,157 |
| 65–74 | $1,664 | $9,529 | $18,164 | $15,943 | $23,609 | $41,244 |
| 75-84 | $1,512 | $12,856 | $18,037 | $14,823 | $19,683 | $28,120 |
| 85+ | $1,394 | $16,301 | $19,288 | $12,682 | $18,574 | $19,085 |
| Women |  |  |  |  |  |  |
| <55 | $1,832 | $1,161 | $17,136 | $18,009 | $27,279 | $26,761 |
| 55–64 | $1,520 | $2,090 | $16,349 | $16,345 | $22,825 | $43,649 |
| 65–74 | $1,595 | $5,106 | $17,238 | $19,159 | $21,705 | $24,802 |
| 75-84 | $1,564 | $13,137 | $17,360 | $17,416 | $20,647 | $33,966 |
| 85+ | $1,670 | $19,679 | $16,545 | $10,656 | $14,854 | $14,675 |
| * Cost per prevalent case of disease.  ** Cost per incident case of disease.  NB. Costs are in Australian dollars, adjusted to the year 2003. | | | | | | |

**Cost-effectiveness modelling input parameters**

The values of all modelling input parameters, including related sources and assumptions, are presented in Table 4 (unit costs) and Table 5 (recruitment parameters).

Table 4: Unit cost parameters.

| **Parameter** | **Distribution** | **Mean (SD)** | **Sources & assumptions** |
| --- | --- | --- | --- |
| **Education/counseling resources** | | | |
| Dietitian hourly rate | gamma | $26.51 ($2.65) | PO2.4 to PO3.4 (i.e. min of 4 years experience) District Health Services Employees' Award - State, QLD Health Professional Stream Wage Rates (http://www.health.qld.gov.au/industrial_relations/wage_rates_professional.asp). |
| Length of individual dietitian visit | gamma | 45 (4.5) | Estimate of minutes per session. Standard deviation assumed to be 10% of point-estimate. |
| Number in group session | gamma | 20 (2.0) | Estimate of 20 people per session. Standard deviation assumed to be 10% of point-estimate. |
| Length of group session | gamma | 60 (6.0) | Estimate of minutes per session. Standard deviation assumed to be 10% of point-estimate. |
| GP delivery cost | *–* | $2.01 | Medicare Benefits Schedule (Nov 2003) [10]. Level B; per minute rate. |
| Peer educator hourly rate | gamma | $15.18 ($1.52) | Entry level AO1/AO2. Administrative Stream - Wage Rates, http://www.health.qld.gov.au/hrpolicies/wage_rates/admin.asp |
| On-costs loading | triangular | 1.6 (0.04) | Includes admin assistance (e.g. with phone calls), stationery & travel. |
| **Community-based activities** | | | |
| Community program (Ashfield-Watt 2007) | gamma | $1.46 ($0.84) | Mean and standard deviation of five UK pilot programs (Sandwell [11], Somerset [12], Airedale and Craven [13], County Durham [14], Hastings [15]). |
| **Cafeteria/supermarket promotion or changes** | | | |
| Number of fresh fruit and vegetable labels | gamma | $141.00 ($14.10) | Number of available fresh fruits (36) and vegetables (105) (www.colesonline.com.au). |
| Length of food/cooking demonstrations | gamma | $60.00 ($6.00) | Estimate of 1 hour. Standard deviation assumed to be 10% of point-estimate. |
| Time spent handing out flyers dressed as large vegetable | gamma | $120.00 ($12.00) | Estimate of 1 person per store for 2 hours on 1 occasion. Standard deviation assumed to be 10% of point-estimate. |
| Cost of fruit/vegetable costume hire | gamma | $110.00 ($11.00) | Cost for hire at Sydney Markets (http://www.freshforkids.com.au/ffk_characters/hire_ffk_characters_suits.html). |
| Cooking demonstrations and workshops | gamma | $453.36 ($45.34) | Average cost, including food, for 30 people over 1-1.5 hours (Nutrition Australia, ACT Healthy Living Schools Program, 2009). |
| Canteen review:  Initial  Follow-up | gamma  gamma | $281.99 ($28.20)  $216.92 ($21.69) | Cost of initial visit and report, and return visit to assess implementation of recommendations (Nutrition Australia, ACT Healthy Living Schools Program, 2009). |
| **Monetary incentives/coupons** | | | |
| Vegetable discount voucher (Kristal 1997) | *–* | $0.86 | Derived from intervention study [16]. |
| Farmers' market discount voucher (Herman 2008a) | *–* | $2.75 | Derived from intervention study [17]. |
| Supermarket discount voucher (Herman 2008b) | *–* | $1.37 | Derived from intervention study [17]. |
| **Telephone & mail delivery resources** | | | |
| Local call | *–* | $0.22 | Telstra business rates, www.telstra.com.au/business. |
| STD call | *–* | $0.30 | Telstra business rates, www.telstra.com.au/business. |
| Mobile flagfall | *–* | $0.30 | Telstra business rates, www.telstra.com.au/business. |
| Mobile per minute rate | *–* | $0.35 | Telstra business rates, www.telstra.com.au/business. |
| Proportion of local/STD/mobile calls | *–* | $0.33 | Assume 1/3 local, 1/3 STD and 1/3 mobile. |
| Length of intervention phone call | gamma | 15 (1.5) | Estimate of 15 minutes per call. Standard deviation assumed to be 10% of point-estimate. |
| Length of phone call (Stevens 2002, 2003) | gamma | 7.5 (0.75) | Mean call of 5-10 minutes; assume. Standard deviation is 10% of point-estimate. |
| Length of phone call (Heimendinger 2005) | gamma | 14 (1.4) | Mean call of 14 minutes; assume. Standard deviation is 10% of point-estimate. |
| Length of phone call (reminder call only) | gamma | 2.0 (0.2) | Estimate of 2 minutes per call. Standard deviation assumed to be 10% of point-estimate. |
| Length of phone call (Kristal 2000) | gamma | 18 (1.8) | Mean call of 15-20 minutes; assume. Standard deviation is 10% of point-estimate. |
| Regular parcel delivery | *–* | $3.64 | Cost of regular parcel delivery (1-250g); Australia Post rates. |
| Large letter postage | *–* | $0.92 | Cost of A4 letter delivery; Australia Post rates. |
| Regular letter postage | *–* | $0.43 | Cost of postage paid letter (DL size); Australia Post rates. |
| Envelope (DL) | gamma | $0.07 ($0.01) | DL black & white envelope; unit price per 1000; http://www.theonlineprinter.com.au/quote_dis_staenvdl.aspx |
| Envelope (C5) | gamma | $0.13 ($0.01) | C5 black & white envelope; unit price per 1000; http://www.theonlineprinter.com.au/quote_dis_staenvc5.aspx |
| Envelope (C4) | gamma | $0.15 ($0.01) | C4 black & white envelope; unit price per 1000; http://www.theonlineprinter.com.au/quote_dis_staenvdl.aspx |
| **Printed information & promotional material** | | | |
| letterhead | gamma | $0.09 ($0.01) | A4 colour; unit price per 1000; http://www.theonlineprinter.com.au/quote_dis_staltra4.aspx |
| newsletter | gamma | $0.49 ($0.05) | Per unit cost of printing and file processing for A4 colour booklet (The Online Printer; http://www.theonlineprinter.com.au/quote_dis_booka4.aspx); deflated. |
| recipe book/calendar | gamma | $0.43 ($0.04) | 12 page A5 gloss colour booklet; unit price per 1000 ; http://www.theonlineprinter.com.au/quote_dis_booka5.aspx |
| manual | gamma | $2.05 ($0.20) | 40 page A4 gloss colour booklet; unit price per 1000 ; http://www.theonlineprinter.com.au/quote_dis_booka4.aspx |
| brochures | gamma | $0.08 ($0.01) | 6 page gloss colour z-fold rackcard/flyer; unit price per 1000; http://www.theonlineprinter.com.au/quote_dis_crdrakpg6.aspx |
| posters A3 | gamma | $0.22 ($0.02) | 1-sided A3 gloss colour poster; unit price per 500; http://www.theonlineprinter.com.au/quote_dis_psta3.aspx |
| posters A2 | gamma | $0.27 ($0.03) | 1-sided A2 gloss colour poster; unit price per 250; http://www.theonlineprinter.com.au/quote_dis_psta2.aspx |
| food labels/signs | gamma | $0.06 ($0.01) | 'Indoor' business card-sized sticker/label; unit price per 1000; http://www.theonlineprinter.com.au/quote_dis_sticker_bc.aspx |
| logo magnets | gamma | $0.22 ($0.02) | cost per magnet; unit price per 1000 ; http://www.theonlineprinter.com.au/quote_dis_magnet_fridge.aspx |
| logo pencils | gamma | $0.18 ($0.02) | white printed pencil with rubber and logo; unit price per 1000; http://www.paylesspromotions.com.au/PROMOTIONAL-PRODUCTS-Pens-/-Writing-Instruments-Pencils/c52_30_180/index.html |
| Poster display coverage | gamma | 0.1 (0.01) | Estimate mean of one poster for each 10 employees in worksite. Standard deviation assumed to be 10% of point-estimate. |
| **Project management** | | | |
| Number of participants per project officer (Ashfield-Watt 2007) | *–* | 50,000 | Estimate based on five UK pilot programs (Sandwell [11], Somerset [12], Airedale and Craven [13], County Durham [14], Hastings [15]). |
| Number of participants per board member (Ashfield-Watt 2007) | *–* | 20,000 | Estimate based on five UK pilot programs (Sandwell [11], Somerset [12], Airedale and Craven [13], County Durham [14], Hastings [15]). |
| Hours per week (project officer) | *–* | 38 | Estimate of hours spent on advisory board for worksite interventions. |
| Hours per month (board member) | *–* | 1 | Estimate of hours spent on advisory board for worksite interventions. |
| Project officer hourly rate | gamma | $31.81 ($3.18) | PO3.4 to PO4.4 (i.e. min of 4 years experience) District Health Services Employees' Award - State, QLD Health Professional Stream Wage Rates (http://www.health.qld.gov.au/industrial_relations/wage_rates_professional.asp). |
| On-costs loading | triangular | 1.3 (0.04) | Includes administrative assistance (e.g. with phone calls), stationery & travel. |
| Average weekly earnings | *–* | $904.30 | Average Weekly Earnings -seasonally adjusted [18]. |
| Average working hours in a day | *–* | 8 | Derived from average weekly hours worked by full-time workers [19], assuming 5 day working week. |
| **Recruitment & training** | | | |
| Cost of mail delivery per respondent | gamma | $43.47 ($18.31) | Mean and variance of cost in preventive health trials (Garrett et al 2000 [20]; Bjornson-Benson et al 1993 [21]; Gren et al 2009 [22]; Robinson et al 2007 [23]). |
| Cost of mass media per respondent | gamma | $52.48 ($33.64) | Mean and variance of cost in preventive health trials (Garrett et al 2000 [20]; Bjornson-Benson et al 1993 [21]; Gren et al 2009 [22]; Robinson et al 2007 [23]). |
| Cost of GP mail-out | gamma | $159,341 ($15,934) | Cost of national mail-out to GPs for National Pneumococcal Vaccination Programs [24]. |
| **Time & travel** | | | |
| GP time cost | *–* | $109.36 | Hourly rate derived from reimbursement rate for Inner Eastern Melbourne Division of General Practice (www.iemdgp.com.au) for 2003. |
| Patient time cost | *–* | $17.44 | Hourly rate derived from labour force participation[19] and average weekly earnings[18]. |
| Mean patient travel time to and from venue | *–* | 15 | Estimate of minutes. |
| Average cost per trip to and from venue | *–* | $7.45 | Based on average distance travelled to GP for urban (estimate), regional [25] and remote [26] populations, and Royal Automobile Club Victoria private vehicle reimbursement rate for medium 2-3 L vehicles. |
| Average waiting time for dietitian | *–* | 15 | Estimate of minutes. |
| Average waiting time for GP | *–* | 30 | Estimate of minutes. |
| NB. All costs are converted to Australian dollars for the year 2003 using the relevant Australian Health Price Index [9], Consumer Price Index [27] and/or Purchasing Power Parities [28]. | | | |

Table 5: Recruitment parameters.

| **Parameter** | **Distribution** | **Mean (SD)** | **Sources & assumptions** |  |
| --- | --- | --- | --- | --- |
| **General population** | | | | |
| Number of calls to the Cancer Council helpline each year | gamma | 150,000 (15,000) | Estimate of national annual calls (www.cancer.org.au//aboutus/whatwedo.htm). |  |
| Population residing in community:  55-64 years  65-74 years  75-84 years  85+ years | *–*  *–*  *–*  *–* | 99%  98%  93%  70% | Derived, by age, from population proportion not usually residing in non-private dwelling [29]. |  |
| People per household | *–* | 2.5 | Average number of people per household in Australia [30]. |  |
| Response rate from mail delivery | gamma | 2.5% (2.0%) | Mean and variance in response rate reported in preventive health trials (Garrett et al 2000 [20]; Bjornson-Benson et al 1993 [21]; Gren et al 2009 [22]; Robinson et al 2007 [23]). |  |
| Response rate from mass media | gamma | 1.8% (2.1%) | Mean and variance in response rate reported in preventive health trials (Garrett et al 2000 [20]; Bjornson-Benson et al 1993 [21]; Gren et al 2009 [22]; Robinson et al 2007 [23]). |  |
| Recruitment rate (Greene 2008) | gamma | 14% (1.4%) | Recruitment via mass media, phone and mail derived from intervention study [31]. Standard deviation assumed to be 10% of point-estimate. |  |
| Proportion of 21-50 year old women with ≥25% of energy from fat or ≤5 serves/day of FV (Radakovich 2006) | *–* | 93% | Derived from data in the Australian Diabetes and Lifestyle Study [32]. |  |
| Proportion of respondents eligible for intervention (Radakovich 2006) | *–* | 16% | Derived from intervention study [33]. |  |
| Proportion of respondents eligible for intervention (Howard 2006) | *–* | 13% | Derived from intervention study [34]. |  |
| **Supermarket** | | | | |
| Supermarket participation | gamma | 60% (6.0%) | Market share of Coles and Woolworths supermarkets [35]. Standard deviation assumed to be 10% of point-estimate. |  |
| Proportion of supermarket shoppers who do not buy fruits or vegetables in supermarket | gamma | 30% (3.0%) | Nielsen Australia [36] (assume same proportion for vegetables). Standard deviation assumed to be 10% of point-estimate. |  |
| Number of supermarkets | gamma | 1,530 (153) | Number of Coles (750) and Woolworths (780) stores over 1000m^2^ in Australia [35]. Standard deviation assumed to be 10% of point-estimate. |  |
| **Worksite** | | | | |
| Number of worksites | gamma | 60,052 (6,005) | Number of businesses in Australia in 2006-07, operational for more than 4 years, with between 20 and 199 employees [37]. |  |
| Number of workers per worksite | triangular | 80 (42) | Distribution of number of workers per worksite assumed to be triangular with minimum of 20 and maximum of 199 employees. |  |
| Worksite participation | gamma | 34% (5.1%) | Participation in a non-government workplace health promotion nutrition program (2004 National Worksite Health Promotion Survey [38]). |  |
| Worker participation | gamma | 11% (21%) | Derived from systematic review of participation determinants in worksite health promotion programs [39]. |  |
| **Health care setting** | | | | |
| Proportion of population with private health insurance | *–* | 45% | Proportion at 31 March 2009 (Private Health Insurance Administration Council; www.phiac.gov.au/statistics/membershipcoverage/table1.htm). |  |
| Proportion of respondents eligible for intervention (Kristal 2000) | *–* | 72% | Derived from intervention study [16]. |  |
| Proportion of eligible respondents willing to participate (Kristal 2000) | *–* | 67% | Derived from intervention study [16]. |  |
| Proportion of females who have had a cholesterol check in previous 3 years (Stevens 2003) | *–* | 48% | Derived from data in the Australian Diabetes and Lifestyle Study [32]. |  |
| Proportion who have a cholesterol level of at least 5.5 mg/l (Stevens 2003) | *–* | 60% | Derived from data in the Australian Diabetes and Lifestyle Study [32]. |  |
| Number of recruited patients per GP in intervention (Sacerdote 2006) | *–* | 96 | Derived from intervention study [40]. |  |
| GP participation (Sacerdote 2006) | gamma | 33% (9.4%) | Proportion of GPs who are eligible for and receiving Service Incentive Payments for asthma, cervical screening and diabetes programs (Medicare Australia, Practice Incentive Payment statistics; www.medicareaustralia.gov.au/provider/incentives/pip/stats.jsp#N10098). |  |
| Number of GPs | *–* | 16,872 | No. of full time workload equivalent GPs in Australia for 2003-4 [41]. |  |
| **Low income** | | | | |
| Proportion attending maternal/child health centre | gamma | 51% (5.1%) | Mean proportion of children aged 0 to 6 years in Victoria recorded as attending a Maternal and Child Health centre at least once in 2007-08 [42]. Standard deviation assumed to be 10% of point-estimate. |  |
| Proportion eligible and willing to participate (Nitzke 2007) | *–* | 90% | Derived from intervention study [43]. |  |
| Proportion eligible to participate (Havas 2003) | *–* | 80% | Derived from intervention study [44]. |  |
| Proportion willing to participate (Havas 2003) | *–* | 39% | Derived from intervention study [44]. |  |
| Number of peer educators (Havas 2003) | gamma | 1,558 (156) | Estimated from proportion of Child and Youth Health centres (www.cyh.com) in South Australia (population 1,612,000), assuming 1 peer educator per centre. |  |
| Proportion of people approached by peer educators (Havas 2003) | gamma | 90% (9.0%) | Estimate of proportion of participants approached in centres, assuming one peer educator is present in each centre with centres operating an average of one day per week (average of 20 attendees per week). |  |

**References**
