# Supplementary material for: Cost-Effectiveness of Interventions to Promote Fruit and Vegetable Consumption
Source: PLoS One. 2010 Nov 30;5(11):e14148. doi: 10.1371/journal.pone.0014148 (PMC2994753; doi:10.1371/journal.pone.0014148)
Supplement: Text S2 — Intervention descriptions. (0.14 MB DOC) [file pone.0014148.s002.doc]

**Intervention characteristics**

| **Study author & year** | **Country** | **Intervention description** | **Age** | **Sex %female** | **Intervention length (years)** | **Follow-up (months)** |
| --- | --- | --- | --- | --- | --- | --- |
| **General population** |  |  |  |  |  |  |
| Marcus 1998 [1] | United States | Intervention recruits callers to National Cancer Institute's Cancer Information Service. Participants receive telephone counselling and mail-out of tailored documents (booklet, recipe book, brochures, logo magnet and pencil). | 18+ | 80% | 1 | 12 |
| Radakovich 2006 [2] (Nutrition and Breast Health study) | United States | Premenopausal women with >=1 1st-degree relative with breast cancer, in good health with intake of >=25% of energy from fat and <=five servings of FV daily are recruited by mass media. Participants receive fortnightly visit with dietitian until dietary goals met and monthly visits thereafter. | 21-50 | 100% | 1 | 12 |
| Howard 2006 [3] (Women's Health Initiative Dietary Modification Trial) | United States | Postmenopausal women are recruited by mail. Participants receive 3 initial individual counselling sessions, 18 group sessions in year one and 4 group sessions annually from year two to year five. | 50-79 | 100% | 7.5 | 90 |
| Heimendinger 2005a [4] | United States | Intervention recruits callers to the Cancer Information Service. Participants receive tailored booklet by mail. | 18+ | 82% | 1 | 12 |
| Heimendinger 2005b [4] | United States | Intervention recruits callers to the Cancer Information Service. Participants receive one tailored booklet, two newsletters and one letter by mail. | 18+ | 82% | 1 | 12 |
| Heimendinger 2005c [4] | United States | Intervention recruits callers to the Cancer Information Service. Participants receive one tailored booklet, two newsletters and one letter by mail, with re-tailoring of information during the intervention. | 18+ | 82% | 1 | 12 |
| Greene 2008 [5] | United States | Community-dwelling older adults are recruited by mass media, telephone and mail. Participants receive 3 counselling telephone calls, and 3 reports, a manual and newsletters by mail. | 60+ | 73% | 1 | 12, 24 |
| Ashfield-Watt 2007 [6] | United Kingdom | Intervention targets whole population (survey participants randomly selected from electoral role). Local advisory board established to run community events (e.g. school competitions, food tastings, etc.) and work with local retailers to promote fruit and vegetable consumption. | 18+ | 57% | 1 | 12 |
| **Supermarket** |  |  |  |  |  |  |
| Kristal 1997 [7] | United States | Intervention targets all supermarket shoppers (survey participants randomly selected from supermarket cues). Discounted fruit/vegetable items are labelled, and study personnel, dressed as giant vegetables, hand out weekly flyers for 4 months then bi-weekly flyers and discount coupons for 4 months. | 18+ | 84% | 1 | 12 |
| **Worksite** |  |  |  |  |  |  |
| Tilley 1999 [8] | United States, Canada | Employees of automotive worksite offered 5 educational classes, self-help materials (by mail), individualised feedback (in year 2), and 4 annual newsletters. Posters and brochures are made available in worksite. | 18-65 | 3% | 1 | 12, 24 |
| Hebert, 1993 [9] | United States | Intervention is co-ordinated by representative employee advisory board. Intervention includes kick-off event (e.g. taste tests, quiz and prize, presentations, educational material) labelling of cafeteria food with some menu and recipe changes (in consultation with outside food service providers). Employees also offered informational classes, weight management sessions and slide presentations. | 18-65 | Not reported | 1.25 | 15 |
| Sorensen 1996 [10], Glanz 1998 [11] (Working Well Trial) | United States | Intervention is co-ordinated by representative employee advisory board. Intervention includes kick-off event (e.g. exhibits, demonstrations, guest speaker, taste-tests, contest), labelling of foods in cafeteria and vending machines with menu-changes at catered events. Employees also offered group skills classes, brochures, posters and self-help materials. | 18-65 | 28% | 2 | 24 |
| Emmons 1999 [12] (Working Healthy Project) | United States | Intervention is co-ordinated by representative employee advisory board. Intervention includes kick-off event (e.g. exhibits, demonstrations, guest speaker, taste-tests, contest), labelling of foods in cafeteria and vending machines with menu-changes at catered events. Employees also offered group skills classes, brochures, posters and self-help materials. | 18-65 | 48% | 2.5 | 30 |
| Sorensen 1998 [13] (WellWorks Study) | United States | Intervention is co-ordinated by representative employee advisory board. Intervention includes kick-off event (e.g. exhibits, demonstrations, guest speaker, taste-tests, contest), labelling of foods in cafeteria and vending machines with menu-changes at catered events. Employees also offered group skills classes, brochures, posters and self-help materials. | 18-65 | 24% | 2 | 24 |
| Beresford 2001 [14] (Seattle 5 a Day study) | United States | Intervention is co-ordinated by representative employee advisory board. Intervention includes kick-off event, food preparation and cooking demonstrations in cafeteria, labelling of foods, signs, special events (e.g. vegetable soup day) and development of a children’s cookbook. Employees also offered monthly messages via brochures, newsletters and flyers, with additional posters and brochures made available over 12 months. | 18-65 | 58% | 2 | 24 |
| Engbers 2006 [15] (FoodSteps) | Netherlands | Intervention targets government office workers with 6 healthy food buffets in canteen, monthly information labelling of canteen foods, one-off labelling of food in vending machines and an information stand. | 18-65 | 35% | 1 | 12 |
| **Health care setting** |  |  |  |  |  |  |
| Kristal 2000 [16] | United States | Participants randomly recruited from Health Maintenance Organisation member list via an information mail-out and telephone call. Intervention includes one motivational phone call and mail-out of self-help materials and newsletters. | 18-69 | 50% | 1 | 12 |
| Stevens 2003 [17] | United States | Participants recruited from Health Maintenance Organisation list of members with recent (negative) mammogram and cholesterol level >200mg/dl via mail-out. Intervention includes two 45 minute individualised counselling sessions, and two follow-up telephone calls. | 40-70 | 100% | 1 | 12 |
| Sacerdote 2006 [18] | Italy | Healthy men and women recruited during general practitioner (GP) visit. Participants receive 15 minutes of GP counselling and a brochure. Participating GPs attend a 4-day training course delivered by a nutritionist. | 18-65 | 52% | 1 | 12 |
| **Low income** |  |  |  |  |  |  |
| Nitzke 2007 [19] | United States | Low income young adults recruited via advertising and word-of-mouth. Participants receive two educational telephone calls, and six mail-outs of material, including two computer-tailored feedback reports, 6 newsletters and one magazine. | 18-24 | 60% | 1 | 12 |
| Herman 2008a [20] (WIC) | United States | Postpartum women recruited while enrolling for Women, Infants & Children services. Participants receive five $2 vouchers each week for 6 months to spend on produce at the farmers’ markets. | 18+ | 100% | 1 | 12 |
| Herman 2008b [20] (WIC) | United States | Postpartum women recruited while enrolling for Women, Infants & Children services. Participants receive ten $1 vouchers each week for 6 months to spend on produce at the supermarket. | 18+ | 100% | 1 | 12 |
| Havas 2003 [21] (WIC 5-a-day) | United States | Women recruited by ‘peer educators’ while collecting vouchers as part of the Special Supplemental Nutrition Program run by the Women, Infants, and Children services. Participants are offered a 1-day interactive kick-off event and four 45 minute group sessions or five 1-day fairs. They also receive four mail-outs of tailored information and incentives, display posters, coloured, illustrated booklet, recipe book, children's activity book, videotape, refrigerator magnet and calendar reminder sheets, and they receive reminder telephone calls from peer educators. Peer educators receive two days of training before each event. | 18+ | 100% | 1.67 | 20 |

**References**

1. Marcus AC, Heimendinger J, Wolfe P, Rimer BK, Morra M, et al. (1998) Increasing Fruit and Vegetable Consumption among Callers to the CIS: Results from a Randomized Trial. Prev Med 27: S16-S28.

2. Radakovich K, Heilbrun LK, Venkatranamamoorthy R, Lababidi S, Klurfeld DM, et al. (2006) Women Participating in a Dietary Intervention Trial Maintain Dietary Changes Without Much Effect on Household Members. Nutr Cancer 55: 44 - 52.

3. Howard BV, Van Horn L, Hsia J, Manson JE, Stefanick ML, et al. (2006) Low-fat dietary pattern and risk of cardiovascular disease: the Women's Health Initiative Randomized Controlled Dietary Modification Trial. JAMA 295: 655-666.

4. Heimendinger J, O'Neill C, Marcus AC, Wolfe P, Julesburg K, et al. (2005) Multiple Tailored Messages are Effective in Increasing Fruit and Vegetable Consumption Among Callers to the Cancer Information Service. Journal of Health Communication: International Perspectives 10: 65 - 82.

5. Greene G, Fey-Yensan N, Padula C, Rossi S, Rossi J, et al. (2008) Change in fruit and vegetable intake over 24 months in older adults: results of the SENIOR project intervention. The Gerontologist 48: 378-387.

6. Ashfield-Watt PAL, Welch AA, Godward S, Bingham SA (2007) Effect of a pilot community intervention on fruit and vegetable intakes: use of FACET (Five-a-day Community Evaluation Tool). Public Health Nutr 10: 671-680.

7. Kristal AR, Goldenhar L, Muldoon J, Morton RF (1997) Evaluation of a supermarket intervention to increase consumption of fruits and vegetables. American journal of health promotion : AJHP 11: 422-425.

8. Tilley BC, Glanz K, Kristal AR, Hirst K, Li S, et al. (1999) Nutrition Intervention for High-Risk Auto Workers: Results of the Next Step Trial,. Prev Med 28: 284-292.

9. Hebert JR, Stoddard AM, Harris DR, Sorensen G, Hunt MK, et al. (1993) Measuring the effect of a worksite-based nutrition intervention on food consumption. Ann Epidemiol 3: 629-635.

10. Sorensen G, Thompson B, Glanz K, Feng Z, al e (1996) Work site-based cancer prevention: Primary results from the Working Well Trial. Am J Public Health 86: 939.

11. Glanz K, Patterson RE, Kristal AR, Feng Z, Linnan L, et al. (1998) Impact of work site health promotion on stages of dietary change: the Working Well Trial. Health Educ Behav 25: 448-463.

12. Emmons K, Linnan L, Shadel W, Marcus B, Abrams D (1999) The Working Healthy Project: A Worksite Health-Promotion Trial Targeting Physical Activity, Diet, and Smoking. J Occup Environ Med 41: 545-555.

13. Sorensen G, Stoddard A, Hunt MK, Hebert JR, al e (1998) The effects of a health promotion--health protection intervention on behavior change: The WellWorks study. Am J Public Health 88: 1685.

14. Beresford SAA, Thompson B, Feng Z, Christianson A, McLerran D, et al. (2001) Seattle 5 a Day Worksite Program to Increase Fruit and Vegetable Consumption. Prev Med 32: 230-238.

15. Engbers L, van Poppel M, Chin A Paw M, van Mechelen W (2006) The effects of a controlled worksite environmental intervention on determinants of dietary behavior and self-reported fruit, vegetable and fat intake. BMC Public Health 6: 253.

16. Kristal AR, Curry SJ, Shattuck AL, Feng Z, Li S (2000) A Randomized Trial of a Tailored, Self-Help Dietary Intervention: The Puget Sound Eating Patterns Study. Prev Med 31: 380-389.

17. Stevens VJ, Glasgow RE, Toobert DJ, Karanja N, Smith KS (2003) One-year results from a brief, computer-assisted intervention to decrease consumption of fat and increase consumption of fruits and vegetables. Prev Med 36: 594-600.

18. Sacerdote C, Fiorini L, Rosato R, Audenino M, Valpreda M, et al. (2006) Randomized controlled trial: effect of nutritional counselling in general practice. Int J Epidemiol 35: 409-415.

19. Nitzke S, Kritsch K, Boeckner L, Greene G, Hoerr S, et al. (2007) A Stage-tailored Multi-modal Intervention Increases Fruit and Vegetable Intakes of Low-income Young Adults. Am J Health Promot 22: 6-14.

20. Herman DR, Harrison GG, Afifi AA, Jenks E (2008) Effect of a Targeted Subsidy on Intake of Fruits and Vegetables Among Low-Income Women in the Special Supplemental Nutrition Program for Women, Infants, and Children. Am J Public Health 98: 98-105.

21. Havas S, Anliker J, Greenberg D, Block G, Block T, et al. (2003) Final results of the Maryland WIC food for life program. Prev Med 37: 406-416.
