# Supplementary material for: Cost-Effectiveness of Interventions to Promote Fruit and Vegetable Consumption
Source: PLoS One. 2010 Nov 30;5(11):e14148. doi: 10.1371/journal.pone.0014148 (PMC2994753; doi:10.1371/journal.pone.0014148)
Supplement: Text S3 — Intervention resource cost components. (0.14 MB DOC) [file pone.0014148.s003.doc]

Table 1 Intervention resource cost components per participant, by setting.

|  | **Individualized counselling** | **Peer education counselling** | **Workshop or speaker** | **Counseling phone call** | **Tailored documents** | **Non-tailored documents** | **Nutrition displays** | **Cafeteria promotion or choices** | **Community-based activities** | **Family involvement** | **Advisory board** | **Monetary incentives or coupons** | **Staff training** | **Recruitment** | **Time** | **Travel** | **Total cost per participant** |
| --- | --- | --- | --- | --- | --- | --- | --- | --- | --- | --- | --- | --- | --- | --- | --- | --- | --- |
| **General population** |  |  |  |  |  |  |  |  |  |  |  |  |  |  |  |  |  |
| Marcus 1998 (35) |  |  |  | $7 | $5 |  |  |  |  |  |  |  |  | $25 | $4 |  | $42 |
| Radakovich 2006 (37) | $378 |  |  |  |  |  |  |  |  |  |  |  |  | $347 | $249 | $307 | $1,280 |
| Howard 2006 (32) | $60 |  | $45 |  |  |  |  |  |  |  |  |  |  | $332 | $632 | $450 | $1,519 |
| Heimendinger 2005a (30) |  |  |  |  | $2 |  |  |  |  |  |  |  |  | $6 |  |  | $8 |
| Heimendinger 2005b (30) |  |  |  |  | $4 |  |  |  |  |  |  |  |  | $6 |  |  | $10 |
| Heimendinger 2005c (30) |  |  |  |  | $4 |  |  |  |  |  |  |  |  | $6 |  |  | $10 |
| Greene 2008 (25) |  |  |  | $26 | $3 | $15 |  |  |  |  |  |  |  | $699 | $13 |  | $756 |
| Ashfield-Watt 2007 (46) |  |  |  |  |  |  |  |  | $1 |  | $2 |  |  |  |  |  | $3 |
| **Supermarket** |  |  |  |  |  |  |  |  |  |  |  |  |  |  |  |  |  |
| Kristal 1997 (34) |  |  |  |  |  | $30 | $0.004 | $0.18 |  |  |  | $15 |  |  |  |  | $45 |
| **Worksite** |  |  |  |  |  |  |  |  |  |  |  |  |  |  |  |  |  |
| Tilley 1999 (26) |  |  | $7 |  | $1 | $8 |  |  |  |  |  |  |  |  | $107 |  | $122 |
| Hebert, 1993 (45) |  |  | $95 |  |  |  | $15 | $19 |  |  | $1,050 |  |  |  | $1,521 |  | $2,700 |
| Sorensen 1996 (40) |  |  | $3 |  |  | $5 | $15 | $19 |  |  | $155 |  |  |  | $43 |  | $240 |
| Emmons 1999 (43) |  |  | $3 |  |  | $5 | $15 | $19 |  |  | $218 |  |  |  | $43 |  | $303 |
| Sorensen 1998 (39) |  |  | $3 |  |  | $5 | $15 | $19 |  |  | $155 |  |  |  | $43 |  | $240 |
| Beresford 2001 (42) |  |  | $15 |  |  | $6 | $15 | $19 |  | $0.43 | $110 |  |  |  | $27 |  | $192 |
| Engbers 2006 (44) |  |  |  |  |  | $0.10 | $91 | $19 |  |  |  |  |  |  |  |  | $110 |
| **Health care setting** |  |  |  |  |  |  |  |  |  |  |  |  |  |  |  |  |  |
| Kristal 2000 (33) |  |  |  | $9 | $6 | $20 |  |  |  |  |  |  |  | $111 | $4 |  | $202 |
| Stevens 2003 (55) | $40 |  |  | $9 |  |  |  |  |  |  |  |  |  | $186 | $31 | $32 | $297 |
| Sacerdote 2006 (38) | $30 |  |  |  |  | $0 |  |  |  |  |  |  | $39 | $0 | $4 | $21 | $94 |
| **Low income** |  |  |  |  |  |  |  |  |  |  |  |  |  |  |  |  |  |
| Nitzke 2007 (36) |  |  |  | $17 | $2 | $15 |  |  |  |  |  |  |  | $290 | $9 |  | $333 |
| Herman 2008a (31) |  |  |  |  |  |  |  |  |  |  |  | $357 |  |  |  |  | $357 |
| Herman 2008b (31) |  |  |  |  |  |  |  |  |  |  |  | $357 |  |  |  |  | $357 |
| Havas 2003 (29) |  | $2 |  | $5 | $5 | $6 |  |  |  |  |  |  | $70 | $1,249 | $203 | $59 | $1,600 |
| NB. All costs are in Australian dollars, referenced to the year 2003. | | | | | | | | | | | | | | | | | |
