# Supplementary material for: Cost-Effectiveness of Interventions to Promote Fruit and Vegetable Consumption
Source: PLoS One. 2010 Nov 30;5(11):e14148. doi: 10.1371/journal.pone.0014148 (PMC2994753; doi:10.1371/journal.pone.0014148)
Supplement: Text S4 — Evidence classification table. (0.02 MB DOC) [file pone.0014148.s004.doc]

Table 1 System for classification of the strength of evidence underlying the measure of effect for cost-effectiveness analysis [1].

| **Strength category** | **Strength of the evidence** |
| --- | --- |
| “**Sufficient evidence of effectiveness”:** Effectiveness is demonstrated by sufficient evidence from well-designed research. | - The effect is unlikely to be due to chance (e.g. P is < 0.05) and - The effect is unlikely to be due to bias (e.g. evidence from: - a level I study design; - several good quality level II studies; or - several high quality level III-1 or III-2 studies from which effects of bias and confounding can be reasonably excluded on the basis of the design and analysis) |
| **“Limited evidence of effectiveness”:** Effectiveness is demonstrated by limited evidence from studies of varying quality | - The effect is probably not due to chance (e.g. p< 0.05) but - Bias, while not certainly an explanation for the effect, cannot be excluded as a possible explanation (e.g., evidence from: - one level II study of uncertain or indifferent quality; - evidence from one level III-1 or III-2 study of high quality; - evidence from several level III-1 or III-2 studies of insufficiently high quality to rule out bias as a possible explanation; or - evidence from a sizeable number of level III-3 studies which are of good quality and consistent in suggesting an effect). |
| **“Inconclusive evidence of effectiveness”:** Inadequate evidence due to insufficient or inadequate quality research. | - No position could be reached on the presence or absence of an effect of the intervention (eg. no evidence from level I or level II studies and level III studies are available, but they are few and of poor quality, or only level IV studies are available.) |
| National Health and Medical Research Council [2] levels of evidence:  I Evidence obtained from a systematic review of all relevant randomised controlled trials.  II Evidence obtained from at least one properly designed randomised controlled trial.  III-1 Evidence obtained from well-designed pseudo-randomised controlled trials (alternate allocation or some other method).  III-2 Evidence obtained from comparative studies with concurrent controls and allocation not randomised (cohort studies), case-control studies, or interrupted time series with a control group.  III-3 Evidence obtained from comparative studies with historical control, two or more single-arm studies, or interrupted time series without a parallel control group.  IV Evidence obtained from case series, either pre-test or post-test. | |

References

1 Carter R, Stone C, Vos T, et al. Trial of program budgeting and marginal analysis (PBMA) to assist cancer control planning in Australia. Melbourne: Centre for Health Program Evaluation 2000.

2 NHMRC. A guide to the development, implementation and evaluation of clinical practice guidelines. Canberra: National Health and Medical Research Council 1999.
