# Supplementary material for: Cost-Effectiveness of Interventions to Promote Fruit and Vegetable Consumption
Source: PLoS One. 2010 Nov 30;5(11):e14148. doi: 10.1371/journal.pone.0014148 (PMC2994753; doi:10.1371/journal.pone.0014148)
Supplement: Text S5 — Table of undiscounted results. (0.08 MB DOC) [file pone.0014148.s005.doc]

Table 1 Health gain, costs and cost-effectiveness of the interventions to promote fruit and vegetable consumption (undiscounted).

|  | **Mean DALYs averted** | **Proportion of total DALYs** | **Mean intervention cost ($million)** | **Mean disease cost offset ($million)** | **Median CER ($/DALY)** | **Probability (<$50/000/DALY)** |
| --- | --- | --- | --- | --- | --- | --- |
| **General population** |  |  |  |  |  |  |
| Marcus 1998 | 0.39 (0.10 to 0.77) | <0.01% | $0.02 ($0.02 to $0.03) | $0.003 ($0.007 to $0.001) | $48,000 | 53% |
| Radakovich 2006 | 71 (0.73 to 300) | 0.04% | $32 ($0.41 to $130) | $0.59 ($2.5 to $0.01) | $440,000 | 0% |
| Howard 2006 | 140 (11 to 470) | 0.08% | $26 ($2.1 to $85) | $1.1 ($3.8 to $0.08) | $170,000 | 0% |
| Heimendinger 2005a | 0.17 (-0.12 to 0.50) | <0.01% | $0.004 ($0.003 to $0.005) | $0.002 ($0.004 to $0.001) | $14,000 | 76% |
| Heimendinger 2005b | 0.37 (0.08 to 0.76) | <0.01% | $0.005 ($0.004 to $0.007) | $0.003 ($0.007 to $0.001) | $5,200 | 98% |
| Heimendinger 2005c | 0.45 (0.14 to 0.82) | <0.01% | $0.005 ($0.004 to $0.007) | $0.004 ($0.008 to $0.001) | $2,700 | 99% |
| Greene 2008 | 1,100 (260 to 2,100) | 0.64% | $340 ($150 to $620) | $10 ($20 to $2.3) | $290,000 | 0% |
| Ashfield-Watt 2007 | 8,600 (-720 to 19,000) | 5.0% | $47 ($29 to $78) | $75 ($180 to $5.7) | Dominant | 95% |
| **Supermarket** |  |  |  |  |  |  |
| Kristal 1997 | 170 (-2,300 to 2,700) | 0.10% | $150 ($120 to $180) | $1.4 ($23 to $20) | $1,500,000 | 3% |
| **Worksite** |  |  |  |  |  |  |
| Tilley 1999 | 180 (29 to 560) | 0.10% | $66 ($17 to $150) | $1.5 ($4.5 to $0.23) | $360,000 | 0% |
| Hebert, 1993 | 420 (34 to 1,300) | 0.24% | $1,500 ($700 to $2,800) | $3.4 ($11 to $0.24) | $4,100,000 | 0% |
| Sorensen 1996 | 330 (55 to 920) | 0.19% | $130 ($78 to $200) | $2.6 ($7.2 to $0.42) | $440,000 | 0% |
| Emmons 1999 | 990 (170 to 2,700) | 0.57% | $160 ($100 to $240) | $7.9 ($21 to $1.3) | $170,000 | 0% |
| Sorensen 1998 | 230 (17 to 780) | 0.13% | $130 ($78 to $200) | $1.9 ($6.2 to $0.13) | $700,000 | 0% |
| Beresford 2001 | 520 (-43 to 1,800) | 0.30% | $100 ($61 to $170) | $4.1 ($14 to $0.36) | $230,000 | 0% |
| Engbers 2006 | 2,200 (-9,600 to 18,000) | 1.3% | $60 ($21 to $130) | $18 ($140 to $81) | $23,000 | 58% |
| **Health care setting** |  |  |  |  |  |  |
| Kristal 2000 | 37 (2.3 to 140) | 0.02% | $14 ($8.4 to $28) | $0.31 ($1.1 to $0.02) | $490,000 | 0% |
| Stevens 2003 | 30 (2.1 to 100) | 0.02% | $3.4 ($0.25 to $11) | $0.23 ($0.80 to $0.02) | $100,000 | 2% |
| Sacerdote 2006 | 170 (-220 to 680) | 0.10% | $50 ($26 to $80) | $1.4 ($5.6 to $1.8) | $290,000 | 1% |
| **Low income** |  |  |  |  |  |  |
| Nitzke 2007 | 0.54 (-0.11 to 3.0) | <0.01% | $2.2 ($0.02 to $10) | $0.003 ($0.019 to $0.001) | $4,000,000 | 0% |
| Herman 2008a | 53 (16 to 99) | 0.03% | $8.8 ($7.2 to $11) | $0.45 ($0.90 to $0.12) | $160,000 | 0% |
| Herman 2008b | 22 (-13 to 63) | 0.01% | $8.8 ($7.2 to $11) | $0.19 ($0.57 to $0.12) | $400,000 | 0% |
| Havas 2003 | 58 (5.8 to 130) | 0.03% | $150 ($120 to $190) | $0.48 ($1.1 to $0.02) | $2,700,000 | 0% |
| NB. All values are rounded to two significant figures. Costs are in Australian dollars referenced to the year 2003. The 95% uncertainty interval is presented for all disability-adjusted life years (DALYs) and costs. Where cost-effectiveness ratio (CER) is Dominant, the intervention leads to more health and less cost than if no fruit and vegetable intervention is in place. | | | | | | |
